# Telehealth-based diagnostic testing in general practice during the COVID-19 pandemic: an observational study

**DOI:** 10.3399/BJGPO.2021.0123

**Published:** 2022-01-26

**Authors:** Rae-Anne Hardie, Gorkem Sezgin, Chisato Imai, Emma Gault, Precious McGuire, Muhammad Kashif Sheikh, Christopher Pearce, Tony Badrick, Andrew Georgiou

**Affiliations:** 1 Australian Institute of Health Innovation, Macquarie University, Macquarie Park, NSW, Australia; 2 Gippsland Primary Health Network, Traralgon, VIC, Australia; 3 Eastern Melbourne PHN, Box Hill, VIC, Australia; 4 West Gippsland Healthcare Group, Warragul, VIC, Australia; 5 Outcome Health, Blackburn, VIC, Australia; 6 Royal College of Pathologists of Australasia Quality Assurance Programs, St Leonards, NSW, Australia

**Keywords:** general practice, COVID-19, telemedicine, laboratory testing, pathology testing, diagnosis

## Abstract

**Background:**

Since the World Health Organization declared COVID-19 a pandemic on 11 March 2020, health technologies have been rapidly scaled up to ensure access to care. A significant innovation has been telehealth in general practice. Now widespread, it remains unknown how this shift to virtual care has impacted on quality-of-care indicators such as pathology testing and diagnosis.

**Aim:**

To undertake a comparison of telehealth and face-to-face general practice consultations to: identify if there were differences in the proportion of pathology test referrals from 2019–2020; and quantify any change in pathology test collection and follow-up patterns.

**Design & setting:**

Retrospective observational study of routinely collected electronic patient data from 807 general practices across New South Wales (NSW) and Victoria, Australia.

**Method:**

Multivariate generalised estimating equation models were used to estimate the proportion of pathology test referrals for overall, face-to-face, and telehealth consultations. Pathology test follow-up was described through median (and interquartile range [IQR]) time.

**Results:**

Pathology test referrals declined during periods of high COVID-19 cases, falling from 10.8% in February 2020 to a low of 4.5% during the first peak in April. Overall, pathology test referrals were lower for telehealth than face-to-face consultations. Median time between referral and test collection was 3 days (IQR 1–14) for telehealth and 1 day (IQR 0–7) for face to face.

**Conclusion:**

For telehealth to become part of routine care, it is crucial that gaps in functionality, including difficulty in test referral processes, be addressed. Quality improvements supporting care practices will ensure clinicians’ workflows are supported and patients receive diagnostic testing.

## How this fits in

Pathology testing is an essential factor in quality of care delivered by GPs. While it is known that telehealth has been used extensively during the COVID-19 pandemic and will likely continue to be used, it is essential that its quality of care is optimised across different situations and scenarios. This study shines a spotlight on this often-overlooked key indicator of care. It investigates how test referrals via telehealth compared with face-to-face consultations, providing much-needed evidence to inform quality improvement for the future of telehealth.

## Introduction

Early in the COVID-19 pandemic, innovative solutions were quickly needed to address the safety and access concerns faced by both patients and GPs during enforcement of COVID-19 restrictions. Although attending medical appointments was an approved reason for leaving home,^1^ there was nevertheless a severe initial drop in face-to-face visits from March–June 2020. This drop was seen across the states of Victoria and NSW,^2^ including an Australia-wide decline by approximately 25% compared with the previous year.^3^ Studies in the US showed drops close to 60%.^4,5^ The Australian government underpins private practice activity through the Medicare Benefits Schedule (MBS). Apart from limited programmes, telehealth was not funded before the pandemic. Like many other countries,^6^ the COVID-19 response in Australia was rapid. The progressive release of a list of temporary MBS telehealth services item numbers^7–9^ transpired over mere weeks, intending to address the gap in face-to-face consultations in general practice and reduce the risk of community transmission. While telehealth in Australia was previously limited to rural and remote populations for specialist care, requiring a visit to general practice or a hospital,^10–12^ this was different. It was the first widespread rollout of telehealth in general practice, allowing all patients access to remote consultations with their GPs from their homes and enabling GPs to bill for telehealth.

This programme, which was meant to continue only for a few months, has been extended and modified several times.^8^ The Australian government recently announced support for telehealth until at least the end of 2021.^13^ These extensions reflect the considerable importance that access to care via telehealth has had on general practice, with more than 54 million telehealth services completed Australia-wide between 13 March 2020 and 31 March 2021,^13^ and over 40 million of those by GPs.^3^ Telehealth was supported by the rapid introduction of electronic prescribing, but not by other digital workflows.^8^ In the context of the Australian healthcare setting, telehealth encompasses the use of telecommunication technology, including voice or audio (telephone) and video, to provide a medical consultation and patient care in real time.^14^

Referral for pathology testing is an area that has had to quickly evolve to fit clinical workflows during the expansion of telehealth, with workarounds and solutions developed ad hoc. Pathology tests overall dropped significantly at the start of the pandemic^15^ but it is unknown whether consultation type played any role. Blood tests are the most common type of pathology test conducted in Australia. In a survey study of Australian general practices, blood tests (including chemistry and haematology tests) accounted for over three-quarters (75.8%) of pathology tests in 2015–2016.^16^ At the start of the pandemic in Australia, no electronic systems existed to handle ‘e-referrals’. However, pathology companies have since worked to roll out options to support test referrals,^17^ such as submitting a request online using a form, which is accessed through a digital portal, and faxing or emailing a PDF. These techniques involved disruptive workflows. Despite this, there is not yet a harmonised way for GPs to complete pathology referrals digitally, and it is unknown to what degree GPs took up any of the proposed options.

It is known based on the high uptake of telehealth in both Australia^2,18^ and overseas^5,19^ that it has been well used by both patients and practitioners. A survey by the Consumers Health Forum of Australia revealed strong support from responders for telehealth service as of May 2020.^20^ However, there is little known about how aspects of routine care that are usually performed face to face, including test referrals, were managed. Implementing new technology, which would usually take years of planning, was compressed into days or weeks, leaving little time to integrate technologies that mimic tasks from a standard face-to-face workflow, including prescribing and pathology test referrals. Another technology not well integrated into the telehealth workflow is video consultation, which had very poor uptake in general practice,^5^ which was also evident in a US-based outpatient study.^5^ Therefore, despite the benefits of telehealth, certain activities may have been hindered, many of which are related to continuity of care in general practice. A better understanding of these key indicators of care will be crucial if telehealth is to evolve into a tool used in routine care, whether standalone or in parallel to it.

The aim was to undertake a comparison of telehealth and face-to-face general practice visits to: (1) identify if there were any differences in the proportion of pathology test referrals from 2019–2020; and (2) quantify any change in pathology test collection and follow-up patterns.

## Method

### Study data and definitions

Data were obtained from 807 general practices: 461 from NSW, and 346 from Victoria, Australia. The study population comprised all patients who visited participating general practices between January 2019 and December 2020. Non-identifiable patient health data (including sociodemographic characteristics, MBS claims, and pathology laboratory test records) were extracted by the Population Level Analysis and Reporting (POLAR) tool from electronic health records by data custodians Outcome Health^21^ using previously described methods.^22^ Patient demographic characteristics included sex, age (in 5-year age groups), and Primary Health Network (PHN). Australian PHNs are localised health districts, providing targeted healthcare service improvement programmes based on healthcare requirements of their population.^23^

Only MBS items for GP consultations were included, excluding practice nurses and other practitioners. Consultations were restricted to general attendance type consultations. MBS items for consultations (Category 1: Professional attendances) can only be claimed once per day per procedure for a patient. A ‘GP consultation’ was defined as any consultation during which a MBS consultation item was claimed, restricted to at most once per day per patient. Consultations with no MBS items claimed were excluded. GP consultations were categorised as face to face (that is, occurred in the same physical space) or telehealth (that is, occurred through phone or video-conference) based on claimed MBS item numbers (Supplementary Table S1).

The pathology data contains information on test name, Logical Observation Identifiers Names and Codes (LOINC) for laboratorians,^24^ and results. For the study, only pathology requiring a blood test was included. Such tests were identified by using LOINC systems to detect serum, blood, and plasma tests (identified by the terms: ‘Bld’, ‘Ser’, ‘Plas’, ‘Monocyte’, ‘Neutrophil’, ‘Platelets’, ‘RBC’, and ‘WBC’). Test referral date was linked with MBS item claim date to identify GP consultation accompanying a pathology test.

### Statistical analyses

For aim 1, the time trend was described in GP consultations with accompanying blood test referrals from January 2019–December 2020 using a multivariate generalised estimating equation (GEE) with logit link function, calculated separately for face-to-face and telehealth consultations. Postmargin calculation (population average effect) based on the model was used to estimate the weekly GP consultations with blood tests adjusted by patient demographic factors. The model was fitted with a binary outcome for GP consultations with accompanying blood tests (1: had blood test; 0: did not have blood test). Clusters by general practice and patient were formed, with exchangeable correlation structures. Covariates included were age groups, sex, and PHN. Covariates were selected based on their known difference in pathology testing distributions (for example, males and females have differences in laboratory tests such prostate-specific antigen in men, which can present with differences in overall distributions of test results).

For aim 2, the time between test result and follow-up consultation was compared between face-to-face and telehealth consultations with a pathology test (median days and IQR). The time between test referral and collection was compared (median days and IQR), and the proportion of patients with a follow-up consultation within 14 days after a pathology referral was determined, comparing telehealth with face to face (%).

Additionally, in line with aim 2, it was investigated whether telehealth consultations without pathology referrals were more likely to have a follow-up face-to-face consultation within a short timeframe (7 days) compared with face-to-face consultations without pathology referrals. For this analysis, the outcome variable was having a follow-up consultation within 7 days with a pathology test referral, either: (1) any type consultation; or (2) a face-to-face follow-up consultation. The primary predictor was (initial) consultation type and if a pathology test referral occurred during the consultation. Initial consultations with a previous consultation within the last 7 days were excluded, as these consultations could be continuity of care and would saturate the predictor variable. The same modelling approach was followed as the previous analyses to report odds ratios and 95% confidence intervals (CIs). Covariates included were age groups, sex, week, and PHN.

All statistical analyses were conducted on Stata MP (version 15.1).

## Results

### Pathology testing during COVID-19 in Australia

The study sample contained 37 615 626 GP consultations (2019: 17 720 460; 2020: 19 895 166) (Supplementary Figure S1) from 5 491 783 patients (54.3% female). The number of GP consultations from NSW was 15 110 260 (40.2%), and Victoria was 22 505 366 (59.8%).

**Figure 1.**
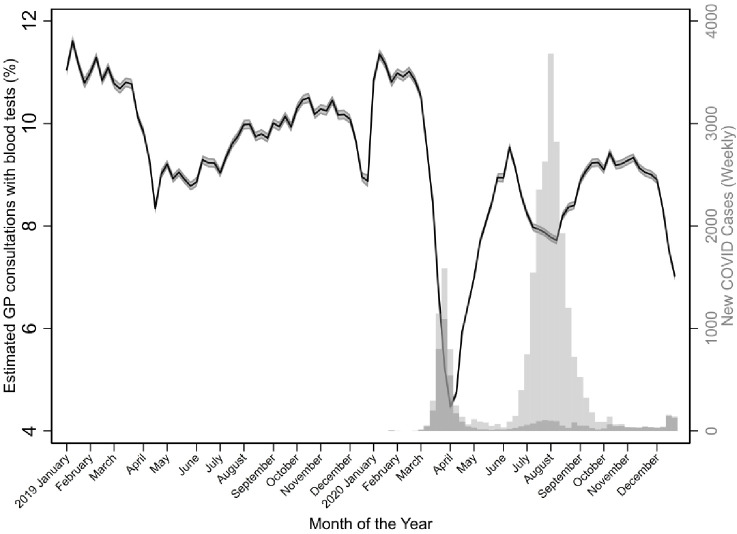
Estimated proportion (and 95% confidence intervals) of GP consultations with blood tests from January 2019–December 2020. Grey bars indicate the number of weekly new COVID-19 cases for Victorian (light grey) and New South Wales (dark grey) PHNs, Australia.

During the study period, 3 356 229 (8.9%) of GP consultations had a blood test (Supplementary Figure S2). During March 2020, Australia’s first COVID-19 peak, blood tests decreased considerably, from an estimated 10.8% in late February, to their lowest, 4.5% in early April, after which pathology testing increased to 9.5% in early June. Another decrease occurred during the following months until early August (7.7%), after which the testing levels increased back to similar levels as 2019 (Figure 1). The dip in December 2020 levels is reflective of the Christmas shutdown period in Australia (a similar dip was observed in 2019).

### Pathology testing in telehealth versus face-to-face consultations

**Figure 2.**
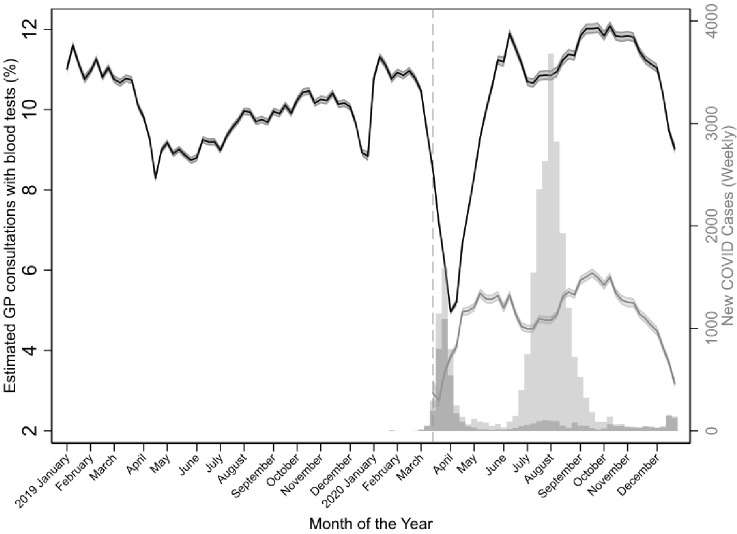
Estimated proportion (and 95% confidence intervals) of face-to-face (black) and telehealth (grey) GP consultations with blood tests from January 2019–December 2020. Dashed vertical line indicates the time at which new telehealth Medicare Benefits Schedule items were first introduced.

A total of 13 459 424 face-to-face and 5 787 639 telehealth (97.5% phone) GP consultations occurred in 2020. Of face-to-face consultations, 1 361 724 (10.1%) had a blood test, and 288,663 (5.0%) of telehealth consultations had a blood test. Blood tests for face-to-face consultations decreased from an estimated 11.0% in late February to 5.1% in early April, after which their levels began to rise to steady levels. Telehealth consultations with a blood test were steady overall, fluctuating between 4% and 6% (Figure 2).

Analysis of proportions of telehealth and face-to-face referred tests for commonly requested blood tests (liver function test [LFT], vitamin D, blood lipids, thyroid function test [TFT], vitamin B12 and folate, haemoglobin A1c [HbA1c], and iron studies [serum ferritin was used as a proxy]) showed similar patterns across all indicators (Supplementary Figures S3-9). The proportion of test referrals during telehealth consultations was approximately half of those compared with face-to-face consultations.

An analysis was also conducted of the differences between the various consultation types (that is, face to face, telephone, telehealth) by the two states, finding no considerable differences other than a slightly higher proportion of consultations with blood tests in Victoria (Supplementary Figure S10).

### Follow-up blood tests

The time between a test result and follow-up consultation was similar for both face-to-face (median 7 days; IQR 4–17 days) and telehealth consultations (median 8 days; IQR 4–19 days). The time taken between a test request and the test collection by a patient was slightly longer for telehealth (median 3 days; IQR 1–14 days) than face-to-face consultations (1 day; IQR 0–7 days). Overall, the proportion of patients who had a follow-up GP consultation within 14 days following the test result was identical for both types of consultations (60.8%). Telehealth consultations without blood tests were 1.31 (95% CI = 1.30 to 1.33; *P*-value <0.001) times more likely to have a follow-up face-to-face consultation with a blood test compared with face-to-face consultations without blood tests within 7 days (Table 1).

**Table 1.** Odds ratios of a consultation having a follow-up consultation with a blood test within 7 days, by consultation type. The initial consultation may either be a face-to-face consultation with or without a blood test, or a telehealth consultation with or without a blood test. The follow-up consultation was reported separately by consultation type: either any kind of consultation or face-to-face consultation, with blood tests. Odds ratios in reference to face-to-face consultations without blood tests

	Follow-up	Any consultation type		Face-to-face consultation	
Initial		Crude OR(95% CI)	Adj OR (95% CI)	Crude OR (95% CI)	Adj OR (95% CI)
Face to face	No blood test	Reference			
	Blood test	3.42(3.40 to 3.45)	3.00 (2.98 to 3.03)	3.28 (3.25 to 3.30)	2.86 (2.84 to 2.89)
Telehealth	No blood test	1.82 (1.81 to 1.84)	1.71 (1.69 to 1.73)	1.35 (1.34 to 1.37)	1.31 (1.30 to 1.33)
	Blood test	3.62 (3.53 to 3.71)	3.13 (3.06 to 3.21)	1.30 (1.25 to 1.36)	1.18 (1.13 to 1.22)

Adj OR = adjusted odds ratio (controlled for age, sex, Primary Health Network [PHN], and week of the year). OR = odds ratio. *P* values are statistically significant (<0.001), unless otherwise specified.

## Discussion

### Summary

A dramatic decline in pathology testing was observed during the start of the COVID-19 pandemic in Australia. The decline was uniform, in general practices across NSW and Victoria. This drop can be attributed to situational barriers, such as the stay-at-home order, temporary pathology laboratory closures, and patient hesitancy about entering medical facilities. Following the release of the MBS telehealth item numbers, however, a large proportion of patients and GPs were seen utilising this new method of consultation. While testing rates appeared to recover for face-to-face consultations, the proportion of telehealth consultations with blood testing was much lower, by about half overall and for most tests that were examined. When a test was requested via telehealth, patients took slightly longer to complete it than via face to face. This may be owing to telehealth patients needing to self-isolate or being more hesitant about visiting a pathology laboratory. This may also be attributed to referral practices taking longer; for example, the time lag for GPs to mail referral forms to patients, fax them to the laboratory, or patients needing to attend the practice to pick up hard copies. Telehealth consultations without pathology referrals were more likely to be followed up by a consultation within 7 days with a blood test referral compared with those following face-to-face consultations. This may be owing to GPs referring patients who were more ill for testing, or requesting patients to come in for other investigations along with pathology referrals. Another factor might be convenience of pathology service to the face-to-face patient, allowing for same-day testing. Similarly, issuing a pathology test referral during telehealth consultations may have technical or logistical barriers, necessitating a follow-up face-to-face consultation.

### Strengths and limitations

Compared with survey-based studies,^25^ the present study is one of the largest, most comprehensive datasets for Australian general practice. Its longitudinal nature and the availability of data allow the observation of changes closer to real time.^21,26^ A strength of using such a dataset curated by data custodians is that data (for example, diagnosis) is coded and non-identifiable, allowing use of many data fields. However, GP free text notes are not visible to researchers (as part of the ethical agreements), owing to potential for identifying features to be revealed. Therefore, researchers did not have access to complete patient records. Consequently, some data could not be analysed. A limitation of electronic patient record data is often lack of standardisation in terminologies (for example, prescriptions, diagnoses, and so on).^27,28^ In the present study, pathology tests were well standardised, largely owing to LOINC codes, resulting in greater accuracy and completeness of test data. Consultation types were based on MBS item numbers, which are also well standardised. Consequently, it was possible to accurately discern consultation types.

A limitation is that despite the authors' best efforts to identify and remove non-blood tests, some may have gone undetected. Another limitation was that only pathology results from fulfilled requests were included, which may underestimate the number of pathology tests requested. Finally, pathology tests may not always be requested or recorded at the same date as a GP consultation. The data linkage between tests and GP consultations were based on dates. Consequently, there may be related pathology tests and GP consultations that were not linked owing to differences in dates. These limitations, however, are not expected to change the conclusions drawn from this study. Finally, only two states, Victoria and NSW, were studied using the available data, therefore the generalisability of the results may be limited. GPs involved in the research were a small subset and GP experience is wide and varied.

### Comparison with existing literature

Given the recent course of the pandemic, there was little published to benchmark the pathology test referral data. One article from Australia looked at the overall uptake of^29^ telehealth earlier in the COVID-19 pandemic based on MBS-subsidised GP consultations.^18^ However, there were no data reported on its use in test referrals or diagnosis. Another Australian study reported telehealth use and GP characteristics during COVID-19; however, it did not include data about test referrals.^25^ There were similar shortfalls in the literature from overseas in reporting the pathology testing via telehealth in primary care settings. Those studies did see similar falls in other service data; for example, an American study demonstrated that despite a lower telehealth uptake rate (4.1% in Q1 2020) than the present study's population,^2,15,29^ reduced blood pressure monitoring and cholesterol level monitoring were associated with telehealth consultations in primary care.^30^

GPs involved with this study proposed several reasons that could potentially explain the lower testing proportions in telehealth consultations. Health information technology infrastructure issues might have partially contributed to reduced testing, making it difficult to get the pathology referral (a form printed by the GP) to the patient who needed it. Most private and public laboratories in Australia prefer a hard copy brought in by the patient. Workarounds, such as GPs faxing forms directly to laboratories, were reported as not working particularly well. There was low uptake or awareness of laboratories’ more recently released digital forms by the GPs contacted in this study. Many patients declined or deferred tests that GPs requested during telehealth consultations, a patient safety issue that could potentially result in delays in diagnosis and treatment. Further research is needed to determine reasons for this, but may include patients’ perceptions that tests were routine and not urgent, or hesitancy about going out during lockdowns. A US-based pre-COVID-19 study showed various factors in patients choosing in-office versus telehealth consultations such as age, race, and sex,^31^ as did another from Australia,^29^ and these, along with potentially a patient’s perception of risk, may also impact a patient’s or GP’s decision on consultation type. Some laboratories closed down temporarily during the height of the pandemic, thus finding a new laboratory that was both nearby and open may have been a barrier to patients who were usually comfortable visiting their local or in-practice laboratories for testing. Finally, there may have been a reduction in 'non-urgent' or patient-requested tests (for example, driven by suggestions from complementary and alternative medicine providers). This deserves further study, as it may indicate a reduction in low-value care. Therefore, both GP and patient-based barriers to accessing pathology testing during the COVID-19 pandemic may have been exacerbated by a lack of integration into telehealth consultations. Further study on these barriers will provide vital evidence to improve referral processes.

### Implications for research and practice

Over the past year, telehealth has developed into a widely used and essential tool for patients and GPs. It is crucial to identify areas that require improvement in the future, especially if a telehealth interface is to be eventually developed and embedded into routine care. The study's findings support the need for enhanced, easier integration of digital pathology referrals within the telehealth consultation. The findings also reveal the need for telehealth support to fit GPs’ workflows, and for pathology laboratories and general practice to work towards harmonisation of test 'e-referrals' technology and infrastructure in a similar way that has allowed e-prescribing to develop.^32^ Future study on outcomes for patients and diseases monitored via telehealth can provide a better understanding of how the future of telehealth in general practice can proceed without compromising quality of care.
